# Assessing available information on the burden of sepsis: global estimates of incidence, prevalence and mortality

**DOI:** 10.7189/jogh.02.010404

**Published:** 2012-06

**Authors:** Issrah Jawad, Ivana Lukšić, Snorri Bjorn Rafnsson

**Affiliations:** Centre for Population Health Sciences, The University of Edinburgh Medical School, Edinburgh, Scotland, UK

## Abstract

**Objective:**

Sepsis is a complex and hard-to-define condition with many different interactions with other disorders. Presently, there are no estimates of the burden of sepsis and septicaemia at the global level and it was not included in the initial Global Burden of Disease study. Non-maternal sepsis has only recently received attention as a substantial global public health problem. The aim of this study was to assess available data on the burden of non-maternal sepsis, severe sepsis and septic shock in the community and to identify key gaps in information needed to estimate the global burden of sepsis.

**Methods:**

Literature review of English language-based studies reporting on the incidence, prevalence, mortality or case-fatality of sepsis, severe sepsis and septic shock. The available literature was searched using the MEDLINE database of citations and abstracts of biomedical research articles published between 1980 and 2008.

**Findings:**

8 studies reported incidence of sepsis, severe sepsis or septic shock at the national level (4 from the USA and 1 from Brazil, the UK, Norway and Australia). No studies on the incidence, prevalence, mortality or case-fatality from sepsis in developing countries were found. The population sepsis incidence ranged from 22 to 240/100 000 (most plausible estimates ranged from 149 to 240/100 000); of severe sepsis from 13 to 300/100 000 (most of the estimates were between 56 and 91/100 000); and of septic shock 11/100 000. Case-fatality rate depends on the setting and severity of disease. It can reach up to 30% for sepsis, 50% for severe sepsis and 80% for septic shock. While the data were compiled using strict inclusion and exclusion criteria, a degree of uncertainty still exists regarding the reported estimates.

**Conclusion:**

The few national-level reports available allow only a very crude estimation of the incidence of sepsis in developed countries while there is apparent lack of data from developing countries. A clear and universal definition of sepsis as well as the development of a sound epidemiological framework to begin addressing the magnitude of this problem is urgently needed through research in developing countries.

Valid and comparable data on the population burden of diseases constitute an essential resource for guiding health policy and informing the process of resource allocation. This is particularly relevant in the developing world, where many diseases demand attention but resources are limited and budgets are tight.

The original Global Burden of Disease (GBD) study was commissioned in 1991 and conducted by the WHO in collaboration with Harvard University and others with the World Bank’s funding. Its purpose was to assess the burden of 107 different diseases and injuries as well as ten risk factors for diseases in 1990 [1]. The uniqueness of the study was reflected in its use of a multitude of sources for producing the estimates and its specially designed new unit - Disability Adjusted Life Year (DALY) – for measuring the actual burden of disease. Soon following its launch, it became clear that information the prevalence and incidence of most diseases and injuries was limited, especially in developing countries [2]. The new GBD study (commenced in the spring of 2007) is the first major effort since the original GBD study was completed to carry out a complete systematic assessment of the data on all diseases and injuries and produce comprehensive and comparable estimates of the burden of diseases, injuries and risk factors for two time periods, 1990 and 2005.

Sepsis is a complex condition with many different interactions with other disorders, and because of this it can be a difficult condition to define. Several medical terms are associated with sepsis, which further complicates diagnosis and identification of the condition. Sepsis is widely defined as a systemic inflammatory response. It has three states, which develop with increased severity: sepsis is followed by severe sepsis, and finally with septic shock (see **Figure 1** for a diagram of the natural history of the sepsis syndrome and **Table 1** for different clinical and epidemiological case definitions of the sepsis syndrome). Presently there are no estimates of the burden of sepsis at the global level and it was not included in the first GBD study. Specifically, while estimates for maternal sepsis are available [5], non-maternal sepsis has only recently been receiving attention as a substantial global problem in terms of morbidity and mortality [3,6]. Sepsis is clearly a problem that has to date been neglected and underestimated by the global health community. It is primarily for this reason that sepsis has been included in the new GBD study.

**Figure 1:**
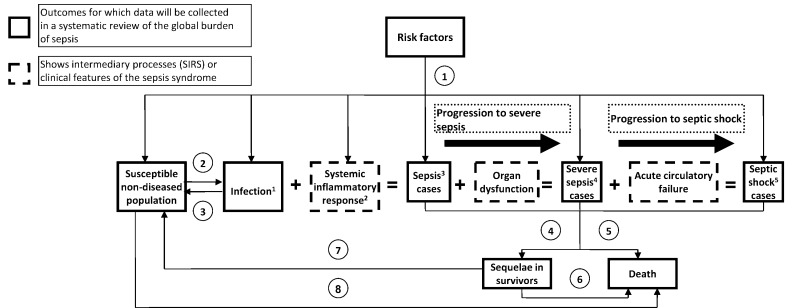
Natural history of sepsis diagram. Key to the diagram: 1) Potentially modifiable risk factors that increase the probability of infection, SIRS and sepsis in a non-diseased population or severe sepsis and septic shock in septic patients; 2) Incidence of sepsis: the rate at which susceptible or exposed individuals become newly affected by sepsis; 3) Remission: the rate at which individuals with sepsis stop being a sepsis case; 4) Sepsis-complication: the rate at which patients experience a complication of sepsis or start to suffer from sequelae of sepsis; 5) Case-fatality (or population mortality rate or relative risk of dying): the rate at which patients die from sepsis; 6) Complication-fatality: the rate at which patients die as a result of a complication of sepsis; 7) Individuals with sequelae who are exposed to the risk factor(s) and are susceptible to acquire infection, SIRS, sepsis, severe sepsis or septic shock again; 8) General mortality: the rate at which the population dies from any condition other than sepsis. Infection has been defined as a pathological process caused by invasion of normally sterile tissue/fluid/body cavity by pathogenic microorganisms; Systemic inflammatory response (SIRS) is a systemic inflammatory state characterized by changes in body temperature, heart rate, respiratory rate and leukocyte blood count; Sepsis is defined as confirmed or suspected infection and SIRS; Severe sepsis is defined as sepsis complicated by organ dysfunction; Septic shock in adults is defined as state of acute circulatory failure characterized by persistent arterial hypotension unexplained by other causes. Paediatric septic shock is defined as tachycardia with signs of decreased perfusion including decreased peripheral pulses, altered alertness, and cool extremities or reduced urinary output. Hypotension occurs later than in adults and is a sign of late and decompensated shock in children [3,4].

**Table 1:** Clinical and epidemiological case definitions of the sepsis syndrome [3,4]

Outcome	Definition	Clinical criteria*	Epidemiological criteria	Relevant ICD-9/10 codes
Infection	Invasion of normally sterile tissue/fluid/ body cavity by microorganisms	Microbiologically confirmed or strongly suspected	*Mortality data:* Relevant ICD codes reported as underlying cause of death (primary code) on a death certificate / *Hospital episode data:* Relevant ICD codes reported as main condition on hospital episode records	*ICD-9:* 001-009, 020-027, 031, 034, 038-041, 098-099, 110-118, 130-136 / *ICD-10:* A00-A09, A20-A28, A31-A32, A39, A42-A49, A54-A64, A65-A69, A70-A74, A75-A79, A90-A99, B35-B49, B50-B64, B95-B97, B99, J00-J39, L00-L08, N39.0
Non-infective causes	Causes of SIRS that are not attributed to infectious agents	Clinically confirmed trauma, thermal injury, or sterile inflammatory processes	*Mortality data:* Relevant ICD codes reported as underlying cause of death (primary code) on a death certificate / *Hospital episode data:* Relevant ICD codes reported as main condition on hospital episode records	*ICD-9:* 574.0, 577.0, 800-904, 910-959, 996-999 / *ICD-10:* J95, K81.0, K85, S00-S99, T00-T14, T20-T32, T33-T35
Systemic inflammatory response (SIRS)	Systemic activation of the innate immune response, regardless of cause	Two or more of the following: temperature >38°C or <36°C; heart rate >90 b/min; respiratory rate >20 b/min or Paco_2_<32 mm Hg; WBC count >12 000/ mm^3^ or <4000/mm^3^ or >10% band forms	*Mortality data:* Relevant ICD codes reported as any (primary or other) cause of death on a death certificate / *Hospital episode data:* Relevant ICD codes reported as main or other condition on hospital episode records	*ICD-9:* 995.90, 995.93
Sepsis	Clinical syndrome defined by the presence of both infection and SIRS	Microbiologically confirmed or strongly suspected infection and two or more of the above (see SIRS clinical criteria; this definition does not reflect the heterogeneity of causes of SIRS/sepsis syndrome, including diverse non-infective causes)	*Mortality data:* Relevant ICD codes reported as any (primary or other) cause of death on a death certificate / *Hospital episode data:* Relevant ICD codes reported as main or other condition on hospital episode records	*ICD-9:* 003.1, 020.2, 038, 630-638, 995.91 / *ICD-10:* A02.1, A09, A22.7, A24.1, A40-A41, A54.8, B37.7, J95.0, T80.2, T81.4, T82.6, T82.7, T83.5, T83.6, T84.5-T84.7, T85.7, T88.0
Severe sepsis	Sepsis complicated by organ dysfunction	Sepsis and organ dysfunction, hypoperfusion or hypotension; hypoperfusion may include: lactic acidosis or oliguria or acute alteration in mental status	*Mortality data:* Relevant ICD codes reported as any (primary or other) cause of death on a death certificate / *Hospital episode data:* Relevant ICD codes reported as main or other condition on hospital episode records	*ICD-9:* 276.2, 286.2, 286.6, 286.9, 287.3-287.5, 293, 348.1, 384.3, 357.82, 359.81, 458.0, 458.8, 458.9, 518.81, 518.82, 518.85, 570, 572.2, 572.3, 580, 584.5-584.9, 585, 780.01, 780.09, 785.5, 785.51, 785.59, 786.09, 796.3, 799.1, 995.92, 995.94 / *ICD-10:* D65, E87.2, G93.4, I.95, I95.1, J96.0, K72, N17, R57.0, T80.2
Septic shock	Circulatory failure characterized by arterial hypotension unexplained by other causes	Sepsis induced hypotension (systolic blood pressure <90 mm Hg or a reduction of ≥40 mm Hg from baseline) despite adequate fluid resuscitation	*Mortality data:* Relevant ICD codes reported as any (primary or other) cause of death on a death certificate / *Hospital episode data:* Relevant ICD codes reported as main or other condition on hospital episode records	*ICD-9:* 009.0, 415.12, 449, 639.5, 785.51, 785.52, 998.0 / *ICD-10:* A41.9, R57.0, T80.2

Producing estimates for the global burden of sepsis as part of the wider GBD study is a complex multi-stage process. The aim of the present study is to determine and evaluate the available information on the burden of sepsis in the community and to identify key gaps in information needed to estimate the global burden of sepsis. This research constitutes a preliminary step in understanding the availability of data on the burden of sepsis and will contribute, alongside other research, to the discussion surrounding whether or not it is possible to assess the global burden of sepsis. The specific objectives of the study are: (i) to undertake a systematic review of the available English language-based literature on the incidence, prevalence and mortality of sepsis, severe sepsis and septic shock; (ii) to apply clear inclusion and exclusion criteria to the data and tabulate the extracted results; (iii) to comment on the quality and spread of data found; and (iv) to discuss the potential future use of this review’s findings and how the results can be further developed.

## METHODS

**Figure 2:**
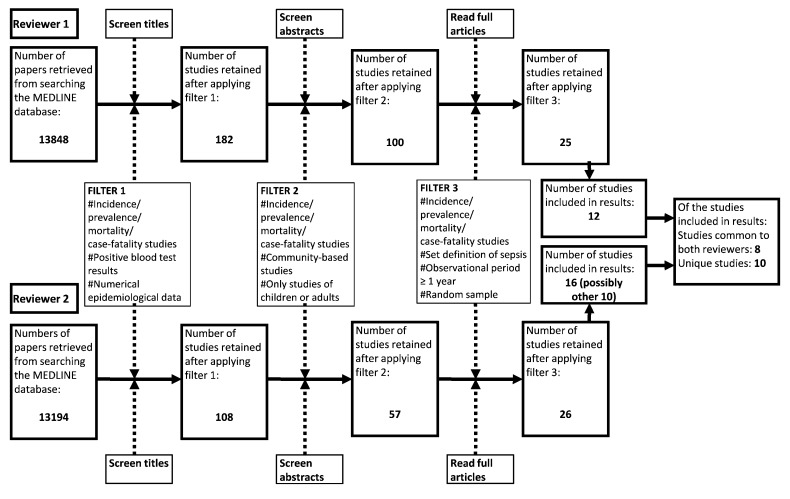
Results from the literature review of the global burden (incidence, prevalence and mortality/case-fatality) of sepsis.

**Figure 2** outlines the research plan that was followed. The literature review was performed by undertaking free text searches in the title and abstract fields of the Medline database for all human studies from 01/01/1980 to 28/02/2008. The following broad search terms were used: ‘sepsis’, ‘septicaemia’/’septicaemia’, ‘incidence’, ‘prevalence’, ‘morbidity’, mortality’, ‘etiology’/‘etiology’ and ‘risk factors’. Both the American and English spellings were used to ensure searches were thorough. The terms sepsis and septicaemia are used interchangeably in the academic literature [7], so both were included in the search and by truncating the word sepsis the search also included studies reviewing septic shock and severe sepsis.

The search also included the terms etiology and risk factors (see above) and while these have no relevance to this study they were included with the wider GBD study in mind. This study provides an opportunity to review this additional data for use in future research on the topic of sepsis. Going through these search results also gives a richer understanding of the subject matter and the pool of information available about sepsis. To ensure the focus on the chosen outcomes, the search term specified that an estimate for incidence, prevalence, mortality or morbidity was included. The initial 13 848 results had their titles reviewed to narrow the number of studies down to those most useful to achieve the aims. The initial screening retained all studies that were relevant to incidence, prevalence, case-fatality, mortality or morbidity, and those that reported any numerical rates in their abstracts as well as any studies that were relevant to the GBD exercise. This resulted in 182 abstracts. This initial filter was very broad to ensure that no potentially useful results were missed and the maximum number of studies possible could enter the next stage of the review.

In the second round of screening, the 182 retained entries were reviewed for reporting a specific figure for incidence, prevalence, mortality or morbidity, which was obtained from their original data, OR whether they were community based studies. At this stage only articles that had their own original data were included. This was done to ensure that methodology behind the estimates reported could be assessed and that the quality of the study could be reviewed. All community studies were included as these are the most helpful type of studies for later projections of the burden to the larger population. At this point the 77 studies that were relevant to the GBD study were separated from the remaining results so as to ensure they were not included in the final filter. For the remaining accepted 100 references, full articles were obtained and reviewed to see if they fulfilled the inclusion criteria or were subject to exclusion criteria (explained in **Figure 2**).

At the final stage there were 12 articles remaining for abstraction from the first reviewer and 16 from the second reviewer, with 8 of them overlapping and further 10 retained after deliberation process (**Figure 2**). The criteria during selection and deliberation were intended to be inclusive but also ensure only good quality studies were included. The requirement for estimates to be from within the date range of 1980 – 2008 was enforced to ensure that the numbers reported were still relevant. The studies had to span at least 12 months to ensure that seasonal variations did not affect the results. The requirement for an acceptable definition of sepsis in the articles was important to ensure that all the studies complied with the ACCP or ICD code definitions of sepsis that were highlighted at the start of this investigation.

Studies that looked at specific population or sepsis as a secondary condition were not included as these cannot be generalized to the whole population. Neonatal and maternal sepsis studies were not included because these are regarded as separate conditions in the ICD classification system.

## RESULTS

The few national-level reports available presented a very broad range of estimates for the incidence of sepsis in different countries. The three available estimates of the incidence of sepsis in the USA, all of which were based on hospital records, ranged from 500 000 [8] to 660 000 cases per year [9]. This roughly translates into an incidence of 300 per 100 000 [10], and represents the most reliable estimate representative of the industrialized countries.

Some of the directly reported estimates of the incidence of sepsis from the smaller regions of Europe include 2007 estimate of 61 per 100 000 person-years in Valencia, Spain [11]; then about 123 per 100 000 per year incidence for hospital admissions due to sepsis in 2006 in France [4], some 38 cases per 100 000 in the adult population in Norway [12] and 149 cases per 100 000 in Finland in 1999 [13].

Much fewer studies are available for the developing world, and the etiological spectrum in low and middle-income countries is likely to be very different from the industrialized ones. Therefore, the burden of sepsis in those parts of the world appears to remain uncharacterized. **Table 2** present the characteristics of identified studies of the incidence, prevalence and mortality rate of sepsis at the population level. All the studies reported their own original results based on hospital findings with a clearly defined credible population denominator which in some cases has been used to produce the national estimates that the studies reported [9,10,13-25].

**Table 2:** Identified studies of the incidence, prevalence and mortality from sepsis

Article	Country studied	Geographic setting	Population studied	Time setting and duration	Incidence as reported (prevalence only where indicated)	Mortality as reported
Martin et al. [9]	United States	Nationwide	750 million hospitalizations in the United States, identified 10 319 418 cases of sepsis	22-year period	240.4 per 100 000 population	17.9% (1995-2000)
Silva et al. [14]	Brazil	Five mixed ICUs in two different regions of Brazil: Săo Paulo State and Santa Catarina State	The total number of enrolled patients was 1383 (81.9%) out of 1688 patients admitted to the ICUs of the participating centers.	21 May 2001 – 31 January 2002	*Sepsis:* 61.4 per 1000 patient-days / *Severe sepsis:* 35.6 per 1000 patient-days / *Septic shock:* 30.0 per 1000 patient-days	*Sepsis:* 33.9% / *Severe sepsis:* 46.9% / *Septic shock:* 52.2%
Elhag et al. [15]	Kuwait	Jabriya, Kuwait City – Mubarak AI-Kabeer Teaching Hospital	3845 patients / 19 606 patients	18 months (January 1982 – June 1983)	10.9/1000 hospital admissions	
Flaatten et al. [13]	Norway	Nationwide	All patients admitted to all Norwegian hospitals during 1999	One year	*National:* 1.49 cases/1000 inhabitants / *Under 1:* 1.1/1000 / *Over 80:* 8.7/1000	13.5%
Hoa et al. [16]	Vietnam	Ho Chi Minh City – southern Viet Nam.	All patients admitted to the hospital whose blood culture was positive	1 June 1993 – 30 May 1994	20.4 episodes per 1000 admissions	6.0%
Harrison et al. [17]	United Kingdom	Nationwide	343 860 admissions to 172 adult units	December 1995 – January 2005	Severe sepsis: 66 hospital admissions per 100 000 population	
Angus et al. [10]	USA	Florida, Maryland, Massachusetts, New Jersey, New York, Virginia and Washington.	All acute care hospitalizations with ICD-9-CM codes for both a bacterial or fungal infectious process	1995 (12 months)	3.0 cases per 1000 population	*Severe sepsis:* 28.6%
Braun et al. [18]	USA	Midwest, Northeast, Southeast, and Western United States	Enrollees in 16 IPA network plans	1 July 1995 – 31 December 1999	Severe sepsis: 0.91 cases of per 1000 enrollees	
Finfer et al. [19]	Australia and New Zealand	Twenty-three closed multi-disciplinary ICUs of 21 hospitals (16 tertiary and 5 University affiliated) in Australia and New Zealand	Results are presented for 3543 ICU admissions in 3338 patients	1999 – 2000	0.77 per 1000 population	
Engel et al. [20]	Germany	Random sample of German hospitals in all 16 federal states of Germany and belonging to 310 hospitals	1380 hospitals (total number of beds: 488727)		*Sepsis prevalence:* 12.4% / *Severe sepsis prevalence:* 11.0%	
Salvo et al. [21]	Italy	99 Italian ICUs, distributed throughout the country	1101 patients who fit criteria from all the ICUs	April 1993 – March 1994		*Sepsis:* 36.0% / *Severe sepsis:* 52.0% / *Septic shock:* 81.8%
Watson et al. [22]	USA	Florida, Maryland, Massachusetts, New Jersey, New York, Virginia and Washington	942 non-federal hospital admissions under 19 y olds.	1995 (12 months)	*Severe sepsis:* 0.56 cases per 1000 children / *Severe sepsis, infants (<1 y):* 5.16 per 1000 / *Severe sepsis, 1–4 y:* 0.49 per 1000 / *Severe sepsis, 5–9 y:* 0.22 per 1000 / *Severe sepsis, 10-14 y:* 0.20 per 1000 / *Severe sepsis, 15–19 y:* 0.37 per 1000	

ICU – intensive care unit, y – year

The search revealed only 8 studies that reported incidence of sepsis, severe sepsis or septic shock at the national level (4 from the USA and 1 each from Brazil, the UK, Norway and Australia) **(****Table 2****)**. Over the period 1980-2008, there appeared to be no studies that assessed incidence, prevalence, mortality or morbidity from sepsis in low-income countries. Reported incidence of sepsis at the population level ranged from 22 to 240/100 000 (although the most plausible estimates were between 149 and 240/100 000); of severe sepsis from 13 to 300/100 000 (although most of the estimates were between 56 and 91/100 000); and of septic shock 11/100 000. Mortality rates depend on the setting and severity of disease. It can reach up to 30% for sepsis, 50% for severe sepsis and 80% for septic shock.

Although the available information is still far from sufficient to understand thoroughly the magnitude of the global burden of sepsis, studies convincingly show that sepsis is a significant health problem even in developed countries. We can deduce that more than 1 in 1000 people in developed countries develop sepsis each year, and between a third and a half of them progress to severe sepsis. Because sepsis is most frequently affecting those most vulnerable (infants, young children and very old and ailing patients), the mortality rate is quite high, even at hospitals: it is about 10% for children, but much higher in the elderly, where it ranges between 15% and 80%, depending on the severity of sepsis and the rate of progression toward septic shock.

Those are the only general conclusions that one can reliably draw from the available literature. The few national-level reports available allow only a very crude estimation of the incidence of sepsis in developed countries. However, there is no information on developing countries. Specific definitions of the problem of sepsis and a sound epidemiological framework to begin addressing the magnitude of this problem are urgently needed through research in developing countries.

## DISCUSSION

The purpose of the GBD study is to quantify measures of what the burden of disease is on a national and international scale in a form that is comprehensive and beneficial for the public health community. The present study can be considered as a preliminary step in an attempt to quantify the disease burden of sepsis, by assessing the availability of data and highlighting any gaps in existing data.

### The definition of sepsis, severe sepsis and septic shock in available studies

A thorough inspection of studies highlights an almost completely consistent definition of sepsis that was concordant with that of the American College of Chest Physicians (ACCP)’s definition (1992) and the ICD codes (version 9) [26]. The ACCP’s definition appears to be the most highly regarded and in several studies the inclusion criteria was simply that they complied with the aforementioned definition [26]. Several studies also referred to the ICD codes as their means of identifying sepsis patients [26]. However, variations in the studies’ definitions of sepsis were only seen in the Latin American studies highlighted by the Jaimes’ (2005) review [27]. The articles deemed all cases where there had been confirmation of bacteremia to be equivalent to a positive diagnosis of septicaemia. This does not comply with the ICD codes or the ACCP’s criteria. In all the studies cases were only included when positive blood cultures had confirmed the presence of bacteria. It was also clearly highlighted whether the study was looking at sepsis, septic shock or severe sepsis and no study combined the three states.

### Study populations and outcomes in available epidemiological studies of the burden of sepsis

Only a limited number of studies examined sepsis or its subsequent states in isolation. Much of the research focused on the epidemiology of sepsis in specific high-risk populations. The majority of available studies assessed post-operative sepsis incidence and burn related sepsis both of which are irrelevant to the aims of this study. The other major target of sepsis research was maternal and neonatal sepsis, an area that has received more attention than non-maternal and non-neonatal sepsis and that was included in the previous GBD Study.

The small number of community-based studies was disappointing; consequently all the studies identified and included were hospital-based. Their use raises questions about how representative the data are. For example, it is possible that such data are less likely to be representative because the rural, and usually poorer, population will be less likely to access these hospitals because of financial or transportation difficulties which results in their exclusion from the hospital population cohort [28]. As well as this limited representation it may also mean that the population denominators the studies state that have been used to calculate their estimates are also inaccurate as a hospital may overestimate how many patients have access to its services. Hospital-based studies only report results from admitted patients and consequently excludes non-admitted sepsis cases. Any amount of misdiagnosis of sepsis patients may affect the reported estimates to an uncertain degree.

Few studies exist on sepsis incidence, prevalence and mortality although several report on the etiology of sepsis; these results show detailed breakdowns of the proportion of septic infections caused by particular bacteria. The comparative abundance of such studies might be explained by their usefulness in developing specific drug vaccines, antibiotics and treatment programmes. In addition, many studies briefly describe the various sequelae associated with sepsis. **Figure 1** highlights the key aspects of the natural history of sepsis.

### Integrity of the results for available epidemiological studies of sepsis

The few included studies inevitably reduce the integrity of the overall results as it becomes more challenging to determine the burden of sepsis from limited data. All efforts were made to try and obtain all the articles that were considered acceptable. Despite this, no incidence or mortality results reported from Africa and only one African study discussed the etiology of sepsis in Nigeria. Similarly, there were no studies from the Asian sub-continent or the rest of Asia, and the geographically closest incidence rates reported for the whole region were from Kuwait. The lack of results means that no clear conclusions could be drawn about sepsis across the world and only figures from isolated countries could be reported.

The few child sepsis studies probably highlight the fact that studies of neonatal sepsis were not included in this review. More reported estimates might be expected for children than adults because historically public health monitoring has been dominated by child health. With increasing child survival in developing countries and more adult deaths then the incidence of sepsis might also become a more sizeable problem. However, such a change in the burden of sepsis is expected to result in an exposure of the inadequacy of data reported for adults.

### Study limitations

The current literature review was limited to studies identified in the Medline databases but could have been extended other databases, including Embase, Web of Science, Global Health Search and the French search engine LILACS, United Nations and WHO databases, non-journal based data as well as any ‘gray literature’ in the form of white papers, un-published research, government reports and working papers. Also, the inclusion of non-English language articles might have increased the completeness of the review. Several non-English papers were excluded and others were not considered due to a lack of an English abstract. In addition, search limitations might also have been reduced and allowed a free text search rather than a title and abstract search. This was not feasible in the present context, however, because of the sheer number of results generated.

### Burden of sepsis estimates and the GBD study

The estimates highlighted in this study can have a valuable impact by themselves as well as a significant impact through the GBD study. In addition to the sepsis incidence and mortality estimates identified, additional research on prevalence and the sepsis severity could be used to then compute them into DALYs, which can help paint a picture of the gross impact of sepsis and not simply just the mortality, prevalence or the incidence in isolation. This is important as it means that sepsis will not just be viewed in terms of how many it kills but it will also include the impact that it has on patients that survive. As can be seen from the natural history diagram (**Figure 1**) there are many sequelae associated with sepsis that can have a life-long disabling impact. Having this knowledge incorporated into the DALYs will help with getting a fuller picture of the impact of sepsis in the community. Such prioritisation may manifest in several ways; it can result in a more significant presence in both regional and global policy and strategy. However, once sepsis is recognized as an important contributor to burden of mortality and ill health, funding agencies may be more likely to consider funding interventions and treatment programmes (eg, vaccine and drug development) as well as investing more in research and development associated with sepsis.

### Recommendations and suggested further research

The next step is to apply the literature review strategy to other databases to ensure that the main sources of relevant data have been considered. This may also mean the inclusion of foreign language articles and having particularly relevant studies translated to ensure that inclusion and exclusion criteria can be applied fully. In order to truly understand the burden of sepsis more time needs to be invested in reviewing non-journal format data including national surveillance data and other ‘gray’ literature available. Specifically, in light of the low results, alternative strategies should be tried as these might yield greater results. One alternative search strategy that needs considering focuses on the specific type of pathogens associated with sepsis. By conducting multiple literature reviews each focusing on a specific pathogen such as meningococcal or typhoid sepsis and gathering estimates for the incidence, prevalence and mortality of each of these forms of sepsis it may be possible then to combine them all for an overall estimate of sepsis.

### Conclusion

Understanding the scale by which sepsis impacts the community is important. The present results show that on average sepsis is reported to have an incidence of 56-91 cases per 100 000 people, with a reported mortality rate of 30%. These estimates are accompanied by wide uncertainty bounds. This indicates that sepsis is a public health problem that the global health community needs to embrace more fully.

The limited results reported in this study highlight the need for greater investment in sepsis research and improved surveillance and reporting of sepsis cases which may also require the development of comprehensive national and international frameworks for data collection and reporting.
